# Is There a Link Between the Pathogenic Human Coronavirus Envelope Protein and Immunopathology? A Review of the Literature

**DOI:** 10.3389/fmicb.2020.02086

**Published:** 2020-09-03

**Authors:** Dewald Schoeman, Burtram C. Fielding

**Affiliations:** Molecular Biology and Virology Research Laboratory, Department of Medical Biosciences, University of the Western Cape, Cape Town, South Africa

**Keywords:** human coronavirus, SARS-CoV, MERS-CoV, SARS-CoV-2, COVID-19, envelope protein, immunopathology

## Abstract

Since the severe acute respiratory syndrome (SARS) outbreak in 2003, human coronaviruses (hCoVs) have been identified as causative agents of severe acute respiratory tract infections. Two more hCoV outbreaks have since occurred, the most recent being SARS-CoV-2, the causative agent of coronavirus disease 2019 (COVID-19). The clinical presentation of SARS and MERS is remarkably similar to COVID-19, with hyperinflammation causing a severe form of the disease in some patients. Previous studies show that the expression of the SARS-CoV E protein is associated with the hyperinflammatory response that could culminate in acute respiratory distress syndrome (ARDS), a potentially fatal complication. This immune-mediated damage is largely caused by a cytokine storm, which is induced by significantly elevated levels of inflammatory cytokines interleukin (IL)-1β and IL-6, which are partly mediated by the expression of the SARS-CoV E protein. The interaction between the SARS-CoV E protein and the host protein, syntenin, as well as the viroporin function of SARS-CoV E, are linked to this cytokine dysregulation. This review aims to compare the clinical presentation of virulent hCoVs with a specific focus on the cause of the immunopathology. The review also proposes that inhibition of IL-1β and IL-6 in severe cases can improve patient outcome.

## Introduction

Coronaviruses (CoVs) (order *Nidovirales*) all have positive sense, single-stranded RNA genomes that range in size between 26 and 32 kb (Gorbalenya et al., 2006; Corman et al., 2018). While they predominantly infect animals, some have, in decades past, been able to cross the species barrier and infect humans. Seven human CoVs (hCoVs) have been identified, of which four – hCoVs 229E, NL63, OC43, and HKU1 – are distributed globally, circulating continuously within the human population, causing mild-to-moderate, self-limiting infections (Su et al., 2016). Conversely, the other three hCoVs, – severe acute respiratory syndrome (SARS)-CoV, Middle East respiratory syndrome (MERS)-CoV, and SARS-CoV-2 – are more virulent and have caused deadly outbreaks during the past two decades (Kahn and McIntosh, 2005; Pyrc et al., 2007; Chafekar and Fielding, 2018).

SARS-CoV caused the first deadly hCoV outbreak in 2003, which was successfully contained in little over 6 months (Hewings-Martin, 2020). The SARS-CoV outbreak resulted in 8096 laboratory-confirmed infections worldwide with 774 deaths, a case-fatality rate of 9.6% (World Health Organization [WHO], 2003). In 2012, the MERS-CoV was identified as the causative agent of MERS in Saudi-Arabia (Broadbent, 2020). The MERS-CoV outbreak of 2012 saw a case-fatality rate of 34.4% from 2499 laboratory-confirmed cases and 861 associated deaths as of December 2019 (World Health Organization [WHO], 2020b). Then, at the end of 2019, SARS-CoV-2 (formerly known as 2019-nCoV) was reported to be responsible for another outbreak of a SARS-like disease in Wuhan, China (CDC, 2020; Gralinski and Menachery, 2020; Kahn, 2020). As of 20 May 2020, 4,789,205 confirmed cases of SARS-CoV-2 infections with at least 318,789 deaths were reported worldwide (World Health Organization [WHO], 2020a).

Undoubtedly, SARS-CoV-2 has an infective profile vastly different from that of the SARS-CoV and MERS-CoV. This is especially evident by the incredibly rapid spread, but much lower case-fatality rate of SARS-CoV-2. The disease associated with the virus was named coronavirus disease 2019 (COVID-19) and is the first hCoV outbreak to be declared a pandemic (World Health Organization [WHO], 2020c,d). This review compares the clinical presentation of the virulent hCoVs, SARS-CoV, and MERS-CoV, to the symptoms reported in COVID-19 patients to date. Evidence is also presented to call attention to the hCoV protein responsible for the immunopathology often seen in severe cases of pathogenic hCoV infections, and how this protein drives the hyperinflammatory response behind this immunopathology. The major inflammatory cytokines involved in this response are highlighted and linked to the inflammatory cytokines reported in COVID-19 patients. Interim potential treatment options that can minimize disease severity, alleviate the burden of disease, and improve patient outcome are proposed while antiviral and vaccine research is still ongoing.

## SARS-CoV and MERS-CoV: A Historical Perspective

SARS- and MERS-CoV cause more severe disease, even in immunocompetent, healthy individuals (Avendano et al., 2003). Patients infected with SARS-CoV present with symptoms resembling atypical pneumonia, exhibiting fever, chills, headache, malaise, myalgia, and dry cough (Lee et al., 2003; Peiris et al., 2003a,b). Those infected with MERS-CoV report similar non-specific symptoms, but demonstrate a much higher case-fatality rate, particularly for elderly persons and those with underlying medical conditions (Assiri et al., 2013a,b; Saad et al., 2014). In some cases, a small proportion of both SARS and MERS patients develop gastrointestinal symptoms (GIT) such as nausea, vomiting, or diarrhea.

The incubation period for SARS is typically between 2 and 7 days, but can be up to 14 days, while for MERS it ranges from 2 to 14 days with a median of approximately 5 days (CDC, 2005, 2019). Unlike the four common hCoVs, the severity of SARS and MERS could likely be attributed to their lack of continuous circulation in the human population. The latter hCoVs had not adapted well to humans as hosts and only managed to cause outbreaks after crossing the species barrier, gaining access to the human population from their animal reservoir through an intermediate host (Perlman and Netland, 2009; Reusken et al., 2013; de Wit et al., 2016).

Patients infected with SARS-CoV and MERS-CoV are at risk of developing acute respiratory distress syndrome (ARDS), a common complication for both viruses. SARS-CoV and MERS-CoV infections have been linked to diffuse alveolar damage (DAD) and are characterized by increased capillary permeability in the lungs, fluid accumulation in the alveoli, coupled with impaired fluid removal mechanisms that culminate in pulmonary edema, inefficient gas exchange, and death (Fowler et al., 2003; Lew et al., 2003; Ng et al., 2016). The incidence of ARDS can be up to 25% in SARS patients, with an associated mortality rate of approximately 50% in these patients (Fowler et al., 2003; Lew et al., 2003). In MERS patients, the incidence of ARDS was less commonly reported, but could develop in 12–20% of patients (Assiri et al., 2013b; World Health Organization Mers-CoV Research Group, 2013). In comparison, some studies have reported that 17–41% of COVID-19 patients had developed ARDS (Chen N. et al., 2020; Wu et al., 2020).

Pro-inflammatory cytokines drive the inflammatory response behind ARDS and are a major contributor to the progression thereof (Channappanavar and Perlman, 2017). Several studies report elevated levels of pro-inflammatory cytokines and chemokines [i.e., interleukin (IL)-1β, IL-6, IL-8, IL-12, tumor necrosis factor α (TNF-α), interferon γ (IFN-γ), CXCL9, CXCL10, CCL2, CCL3, CCL5, granulocyte-macrophage colony-stimulating factor (GM-CSF), and interferon-γ inducible protein 10 kD (IP-10)] associated with the development of ARDS in both SARS and MERS patients (Perlman and Netland, 2009; Totura and Baric, 2012; Channappanavar and Perlman, 2017).

## SARS-CoV-2

A lack of epidemiological and serological information on SARS-CoV-2 currently limits our understanding of COVID-19, but patient data from hospitals in Wuhan have provided some insight into its clinical presentation. Patients exhibit fever, dry cough, myalgia, and shortness of breath with ARDS as a common complication (Chen N. et al., 2020; Huang et al., 2020; Wang D. et al., 2020). A small number of people also developed GIT symptoms (Guan et al., 2020; Guo et al., 2020). Similar to SARS and MERS, the elderly and those with underlying, chronic medical conditions, such as diabetes, hypertension, cardiovascular disease, and chronic obstructive pulmonary disease (COPD) are more prone to serious outcomes; complications associated with ARDS and a cytokine storm, often succumbing to the infection (Guan et al., 2020; Huang et al., 2020). Interestingly, patients who developed ARDS and are admitted to the ICU also have higher levels of inflammatory cytokines, consistent with severe SARS and MERS infections (Lau et al., 2013; Channappanavar and Perlman, 2017; Mahallawi et al., 2018; Chen N. et al., 2020; Huang et al., 2020).

Like SARS and MERS, these cytokines typically include IL-1, IL-2, IL-4, IL-7, IL-10, IL-12, IL-13, IL-17, granulocyte colony-stimulating factor (GCSF), macrophage colony-stimulating factor (MCSF), IP-10, monocyte chemoattractant protein-1 (MCP-1), macrophage inflammatory protein 1-α (MIP-1α), hepatocyte growth factor (HGF), IFN-γ, and TNF-α and, when released collectively in hyperinflammatory conditions, are referred to as a cytokine storm (Chen C. et al., 2020; Huang et al., 2020; Liu et al., 2020; Zhou et al., 2020). Already, several reports have remarked on the clinical similarity between COVID-19, MERS and SARS with respect to its clinical presentation (Chan et al., 2020; Chen G. et al., 2020; Chen T. et al., 2020; Chhikara et al., 2020; Giwa et al., 2020; Huang et al., 2020; Lin et al., 2020; Prompetchara et al., 2020; Rasmussen et al., 2020; Wang D. et al., 2020; Wang Y. et al., 2020; Xie and Chen, 2020; Zhang W. et al., 2020).

The exact cause of this immune-mediated damage, however, remains largely unknown. However, the answer may lie in the mechanics of the viral life cycle and the components that orchestrate it. After all, some viral proteins, especially those involved in pathogenesis, adversely affect the host cell and can be directly implicated in the development of symptoms and, ultimately, the clinical presentation (Manjarrez-Zavala et al., 2013).

## Viral Proteins: At the Expense of the Host

Viruses by their very nature rely entirely on their host cells for replication, propagation, and, ultimately, survival which is achieved by subverting the protein-protein interaction (PPI) networks of their host cells (Gladue et al., 2012; Guth and Sodroski, 2014; Brito and Pinney, 2017). This subversion requires that viruses encode proteins with the necessary motifs to exploit the network of proteins that govern certain host cell processes of benefit to them (Davey et al., 2011; Zheng et al., 2014). The specific motifs, or stretches of peptide sequences, exploited by viruses have received some attention, but, for the most part, have been quite understudied, despite their importance in viral infections. They are grouped into different categories depending on the purpose of the motif and these motifs are employed by several pathogenic viruses to exploit the host cell pathways that can promote the progression of the viral life cycle (Gould et al., 2010; Davey et al., 2011; Sobhy, 2016).

About one-third of the 3′-carboxyl terminus of hCoVs genomes encode for structural proteins as well as additional, so-called accessory proteins (Masters, 2006). While the four structural proteins, spike (S), membrane (M), nucleocapsid (N), and envelope (E), are important for the assembly of a structurally complete virus, the accessory proteins are generally not essential for viral replication *in vitro* (Mortola and Roy, 2004; Masters, 2006; Liu et al., 2014; Wang et al., 2017). While each structural protein has its respective function(s), the E protein is the most enigmatic of them all and is also involved in very important aspects of the coronaviral life cycle. Its involvement in viral assembly is evident by its requirement in the formation of the viral envelope and virus-like particles, while the transmembrane domain (TMD) of E is necessary for the release of viral particles (Corse and Machamer, 2003; Ruch and Machamer, 2011). Of particular relevance to this paper, and the current COVID-19 pandemic, however, is the function of E in the pathogenesis of hCoV infections. Data on the role of E exists predominantly for the prototypic SARS-CoV, which has been studied the most extensively, with some studies for MERS-CoV E.

## E Protein: A Contributor to hCoV Pathogenesis

Effective management and patient care of COVID-19 dictates that we have a better understanding of the disease initiation and progression, or pathogenesis. In the case of virulent viruses, it stands to reason that the natural progression of the viral life cycle would adversely affect the host. These adverse effects inherently give rise to symptoms and, ultimately, manifest clinically. Two documented functions of the hCoV E protein contribute to the pathogenesis of severe hCoV infections.

### The PDZ-Binding Motif (PBM)

All CoV E proteins share the same general architecture (Supplementary Figure S1); a short, hydrophilic amino (N)-terminus, approximately 8–12 residues in length, a subsequent 21–29 residue long hydrophobic region which typically contains two to four cysteine residues, followed by the hydrophilic C-terminus, which accounts for the largest portion of the protein, 39–76 residues in length (Masters, 2006). The last four residues of the C-terminus consists of a motif that allows binding to the postsynaptic density protein 95 (PSD95)/Drosophila disc large tumor suppressor (Dlg1)/zonula occludens-1 protein (zo-1) (PDZ) domain; a domain found in all eukaryotic host cells that functions as a protein-protein recognition sequence to drive host PPIs of significance to viruses (Javier and Rice, 2011). These PDZ domains are found in a multitude of eukaryotic proteins and bind to a specific peptide sequence usually found at the end of the target protein C-terminus (Hung and Sheng, 2002; Gerek et al., 2009). Some viruses, including SARS-CoV, encode proteins with a PDZ-binding motif (PBM) that enables them to exploit the PDZ domains of these host proteins to their advantage (Teoh et al., 2010; Castaño-Rodriguez et al., 2018). This strategy is employed by viruses to modulate various cellular processes including cell-cell junctions, cellular polarity, and signal transduction pathways for the purpose of viral replication, dissemination, and pathogenesis (Javier and Rice, 2011). The terminal portion of the SARS-CoV E protein C-terminus contains a PBM that contributes to its viral pathogenesis and is known to interact with five host proteins (Schoeman and Fielding, 2019). It is classified as a type II PBM, characterized by the consensus sequence X-φ-X-φCOOH, where X represents any amino acid and φ is a hydrophobic residue, usually V, I, or L (Harris and Lim, 2001).

The role of SARS-CoV E in the immune-mediated pathology of severe SARS infections is very well demonstrated by its interaction with the host cell protein, syntenin (Jimenez-Guardeño et al., 2014). Mice infected with recombinant SARS-CoV (rSARS-CoV), containing a fully functional E protein, exhibited lung pathology characterized by severe edema, areas of profuse hemorrhage, and cellular infiltrates. Further analysis showed that the PBM of SARS-CoV E interacted with the PDZ domain of syntenin and triggered an overexpression of pro-inflammatory cytokines that was mediated by the p38 mitogen-activated protein kinase (MAPK) pathway. Expression of pro-inflammatory cytokines IL-1β and IL-6 as well as the acute phase protein serum amyloid A was notably increased. This resulted in an exacerbated immune response toward the infection and the characteristic tissue damage and edema ensued. The infection culminated in ARDS, consistent with severe cases of SARS-CoV infection. Mice infected with rSARS-CoV succumbed to the infection, while all mice infected with rSARS-CoV lacking E (ΔE) survived (Jimenez-Guardeño et al., 2014). Moreover, the authors reported an 80% increase in the survival rate of mice infected with rSARS-CoV when treated with a p38 MAPK inhibitor. This, notably, demonstrates a clear relationship between the pathogenesis and clinical manifestation of SARS-CoV infections, as a direct consequence of the E protein. It also shows that the mortality rate of infected cases can be markedly reduced by limiting the aberrant immune response with a p38 MAPK inhibitor.

So far, the novelty of SARS-CoV-2 has prohibited its complete characterization which makes it challenging to confirm whether the functions of its viral proteins do, in fact, coincide with those already established for other hCoVs, like SARS-CoV. Despite its novelty, SARS-CoV-2 shows a remarkable similarity to SARS-CoV in both clinical and genetic features, making it easier to use our existing knowledge of SARS-CoV to understand SARS-CoV-2 better. Previous reports have remarked that the overall sequence similarity of the E protein among hCoVs is poor (Supplementary Table S1; Ye and Hogue, 2007; Lopez et al., 2008). Still, comparing the E proteins of the pathogenic hCoVs, SARS-CoV, MERS-CoV, and SARS-CoV-2, shows a very high sequence similarity between SARS-CoV E and SARS-CoV-2 E, confirmed by only one other report and supporting the observed clinical similarity between the two hCoVs (Grifoni et al., 2020). This similarity, however, is not shared with the MERS-CoV E protein.

**FIGURE 1:**
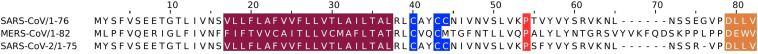
A sequence comparison of the envelope (E) protein amino acid sequences for the pathogenic human coronaviruses (hCoVs). The comparison was constructed using Jalview software (v 2.11.1.0) and the important sequence features transmembrane domain (TMD) (brown), conserved cysteine (blue) and proline (red) residues, and the PDZ-binding motif (PBM) (orange) are indicated. The E protein reference amino acid sequences for SARS-CoV (P59637), MERS-CoV (K9N5R3), and SARS-CoV-2 (QHD43418.1), along with their accession IDs, were obtained from the NCBI database.

A sequence comparison of the virulent hCoV E protein sequences demonstrates that important features such as the topological domains, conserved residues, and the PBM also remain largely intact across these hCoVs (Figure 1). The secondary structure of SARS-CoV E shows that it contains one TMD after a short N-terminus and, based on the similarity between SARS-CoV E and SARS-CoV-2 E having only a four amino acid difference, SARS-CoV-2 E follows the same architecture; one TMD that is most likely in the same location and consists of the same residues (Figure 1). Certain key residues are also conserved, particularly the cysteine residues at positions 40, 43, and 44 (C40, C43, C44), and a proline residue at position 54 (P54) (Figure 1). Cysteine residues adjacent to the TMD of integral membrane proteins, like E, serve as targets for palmitoylation (He et al., 2012). In different CoV E proteins, palmitoylated cysteine residues are important for viral assembly, protein-membrane interaction, and stabilization of the E protein (Boscarino et al., 2008; Lopez et al., 2008). The importance of residues C40, C43, and C44 is, thus, highlighted by their conservation and proximity to the TMD. A chimeric SARS-CoV E protein showed the importance of P54 in the localization of E to the Golgi complex as a chimeric E protein with a mutated P54 residue localized to the plasma membrane instead (Cohen et al., 2011). The conservation of residues C40, C43, C44, and P54 suggest that they might serve similar purposes in SARS-CoV-2 than what they do in SARS-CoV.

The PBM of each hCoV, except MERS-CoV, also consists of at least two definitive hydrophobic residues (V, I, or L), consistent with the consensus sequence for a type II PBM (Figure 1; Harris and Lim, 2001). Only one of the four PBM residues in the PBM of MERS-CoV E is hydrophobic and another (tryptophan) is slightly more hydrophilic than hydrophobic, based on the Kyte and Doolittle (1982) hydropathy table. However, the scarcity of information on hCoV E proteins other than SARS-CoV, makes it difficult to determine the exact reason for this. Nevertheless, the PBMs of SARS-CoV and SARS-CoV-2 are remarkably identical and, given the role of E in SARS-CoV pathogenesis, it supports the similarity in clinical presentation and severity of these two hCoV infections. It also suggests that the SARS-CoV-2 E PBM might interact with syntenin in manner similar to SARS-CoV E. Accordingly, this would allow for treatment strategies and patient care to adopt a more focused approach as the existing data on the SARS-CoV E PBM and its role in SARS pathogenesis would be most beneficial in mitigating the immunopathology often seen in severe COVID-19 cases. Understandably, this sequence similarity merely suggests the existence of a relationship between the similarity of the SARS-CoV and SARS-CoV-2 E protein PBMs and the clinical presentations of these hCoV infections. Although it certainly is noteworthy, experimental evidence is required to corroborate whether this relationship is merely incidental, or whether it could potentially allude to the clinical manifestation or severity of a particular hCoV infection and whether it might be of therapeutic value in COVID-19 patients.

The SARS-CoV E PBM further contributes to viral pathogenesis by its interaction with the PDZ domain of the protein associated with Caenorhabditis elegans lin-7 protein 1 (PALS1) (Teoh et al., 2010). The binding of SARS-CoV E to PALS1, a protein normally associated with tight junctions, redistributed it from the tight junctions of the lung epithelium to the ER-Golgi intermediate compartment (ERGIC) where E assembles. The authors proposed that the redistribution of PALS1 can progressively disrupt tight junctions and contribute to the desquamation of the alveolar wall, creating a breach in the epithelial barrier. This would allow virions to infiltrate the underlying tissues and reach the systemic circulation, disseminating to other organs. Although the study only managed to demonstrate the E-mediated redistribution of PALS1 *in vitro*, the clinical importance of this interaction is consistent with histopathological observations made in lung biopsies obtained from SARS-CoV-infected patients and cynomolgus macaques. The biopsies consistently demonstrated that severe DAD to the lung was accompanied by a massive infiltration of monocytes and macrophages in the alveolar space, a thickened epithelial wall, fused alveolar septa, and hemorrhagic septa with necrotic lesions (Kuiken et al., 2003; Peiris et al., 2003a; Li et al., 2005). Further corroboration comes from studies that show massive recruitment of leukocytes to the site of infection through chemokines and cytokines produced by human airway epithelia, strongly implicating inflammation in the contribution of DAD (Lau and Peiris, 2005; Thiel and Weber, 2008; Channappanavar and Perlman, 2017).

Granted, although this interaction has only been demonstrated in SARS-CoV, it should not diminish the possibility of it occurring in a similar fashion in other virulent hCoV infections such as SARS-CoV-2. It is likely that the PBM of SARS-CoV-2 E can also interact with PALS1 in an analogous manner and cause dissemination of the virus. In fact, the presence of a PBM at the C-terminus of each virulent hCoV indicates that they might all be capable of interacting with host proteins, such as syntenin and PALS1, similar to SARS-CoV. Experimental evidence is, of course, warranted to provide a solid scientific basis, but it would also provide much need valuable insight into why hCoVs clinically manifest in different severities.

A more recent *in silico* study used molecular docking to show that the deletion of glutamic acid 69 (E69) and glycine 70 (G70) residues from SARS-CoV E and the substitution thereof with arginine 69 (R69) in SARS-CoV-2 E enhances binding between SARS-CoV-2 E and PALS1 (De Maio et al., 2020). Acquisition of R69 produced a salt bridge and several hydrogen bonds between E and the PALS1 binding pocket, strengthening the interaction between E and PALS1. In comparison, the small sidechain of G70 prohibited the formation of such bonds between SARS-CoV E and PALS1, reducing the strength of the E-PALS1 interaction. The Gibbs free energy of SARS-CoV-2 E (−97.10 kcal/mol) also showed that it had a higher affinity for PALS1 than SARS-CoV E (−63.62 kcal/mol) did. This data demonstrates that SARS-CoV-2 E could disrupt the pulmonary epithelial barrier and amplify the inflammatory process more effectively than SARS-CoV E does. It could also alter the nature of PPIs with other viral and host proteins.

### Channel Activity and the Inflammasome

The hydrophobic TMD of the E protein is an important component necessary for the assembly of a multimeric structure known as a viroporin; low-molecular-weight proteins that typically contain an amphipathic α-helix and are encoded by many animal viruses. Viroporins oligomerize and can channel various ions, altering the permeability properties of membranes within the host cell. Upon oligomerization, viroporins form a hydrophilic pore that permits the transport of ions across the membrane as the hydrophilic residues face the interior of the pore and the hydrophobic residues face outward toward the phospholipid bilayer (Gonzalez and Carrasco, 2003; Guo et al., 2015). The SARS-CoV E protein viroporin possesses ion-channel (IC) activity and can transport various ions (Na^+^, K^+^, Cl^–^, and Ca^2+^) (Wilson et al., 2004; Nieto-Torres et al., 2015). The importance of this IC property is evident in its contribution to the pathogenesis observed in a SARS infection.

The (NOD)-like receptor protein 3 (NLRP3) inflammasome is a multimeric molecular platform that can be activated by several factors, including increased levels of intracellular Ca^2+^, and contributes to the inflammatory response by stimulating IL-1β production (Allen et al., 2009; Jo et al., 2016). The IC activity of the SARS-CoV E protein has been linked to activation of the inflammasome and disease severity (Nieto-Torres et al., 2014). Mice infected with IC-proficient rSARS-CoV E developed pulmonary edema, lung damage, and succumbed to the infection due to significantly increased levels of inflammatory cytokines IL-1β, IL-6, and TNF-α. Conversely, mice infected with IC-deficient rSARS-CoV E exhibit reduced levels of inflammasome-activated IL-1β, and mice recovered from the infection. The IC activity of SARS-CoV E, therefore, directly correlates with inflammasome activation and an ensuing inflammatory response that causes lung damage. The inflammatory pathology was attributed to a Ca^2+^ imbalance that activated the NLRP3 inflammasome and induced the production of IL-1β (Nieto-Torres et al., 2015). Only two other hCoV E proteins have been shown to possess IC activity: MERS-CoV and hCoV-229E (Wilson et al., 2006; Surya et al., 2015). However, since no experimental evidence exists to link the IC property of either E protein to NLRP3 inflammasome activation, it can only be hypothesized as to whether these hCoVs are equally capable of inducing a pathologic immune response as SARS-CoV does.

**FIGURE 2:**
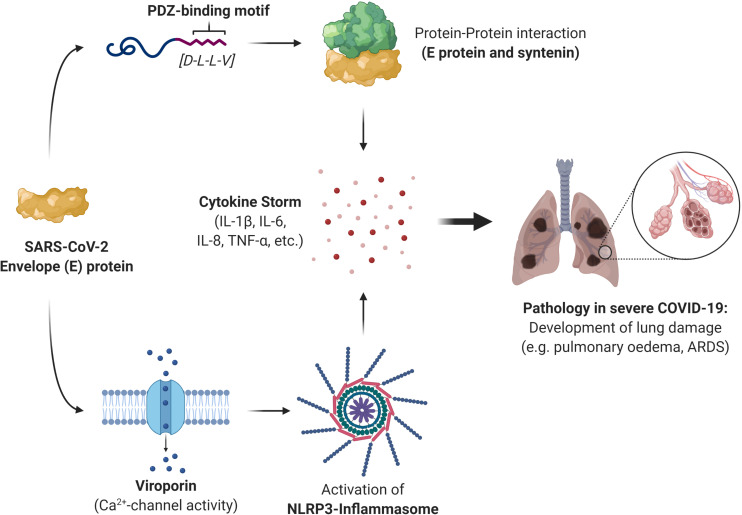
A summary of the role that the severe acute respiratory syndrome coronavirus 2 (SARS-CoV-2) envelope (E) protein plays in the immunopathology of severe coronavirus disease 2019 (COVID-19) cases. The E protein can induce a cytokine storm through protein-protein interaction (PPI) with the host protein syntenin and is mediated by the last four residues of the E protein which constitute the PDZ-binding motif (PBM). This activates the p38 MAPK signaling pathway, triggering the release of inflammatory cytokines. The E protein can also stimulate the release of the inflammatory cytokine interleukin (IL)-1β through its ion-channel (IC) activity. It forms a viroporin that can channel calcium ions (Ca^2+^), which activates the NLRP3 inflammasome that produces IL-1β. The resulting cytokine storm is responsible for the immune-mediated pathology often seen in severe COVID-19 cases and give rise to complications such as pulmonary edema and the acute respiratory distress syndrome (ARDS).

Several other pathogenic viruses also possess viroporin proteins capable of activating the NLRP3 inflammasome; the small hydrophobic (SH) protein of respiratory syncytial virus, influenza virus M2 protein, encephalomyocarditis virus 2B protein, rhinovirus 2B protein, and the hepatitis C virus (HCV) p7 protein (Guo et al., 2015). It is also worth mentioning that a number of viroporin inhibitors have been researched in an effort to inhibit the IC properties of the picornavirus, HCV, SARS-CoV, HIV-1, and influenza A virus. Most inhibitors, however, have exhibited some challenges, including mere moderate inhibition, the formation of resistant variants of viruses, and cytotoxic concentrations, preventing the clinical implementation of such inhibitors (Griffin et al., 2008; Nieva et al., 2012; OuYang and Chou, 2014). Given the challenges faced with these inhibitors, perhaps it would be more prudent to divert the attention toward addressing the fundamental source of viroporins: the viral protein itself. The involvement of the SARS-CoV-2 E protein in the cytokine storm, and the consequent immunopathology of COVID-19, would make the viral protein itself a much more suitable therapeutic target than simply disrupting its PPIs or its IC activity alone. Based on the literature for the pathogenic hCoVs, SARS-CoV and MERS-CoV, we propose that the PBM and IC activity of SARS-CoV-2 E is very likely responsible for the cytokine storm induction and the consequent immunopathology often seen in severe COVID-19 cases (Figure 2).

## Cytokines Il-1β and Il-6 in SARS and COVID-19 Immunopathology

The presence of IL-1β in the pathogenesis and immunopathology of SARS has been well-demonstrated. Interleukin-1β is a potent inflammatory cytokine – the result of a series of cellular signals and stimuli, involving the nuclear factor kappa B (NF-κB) pathway and the NLRP3 inflammasome (Jo et al., 2016). A variety of stimuli is capable of inducing IL-1β production, including products of infectious agents, ionic imbalances inside the cell, exogenous particulates, and molecules associated with cellular damage (Jo et al., 2016). Once released into circulation, IL-1β can cause inflammation and perpetuate the inflammatory response by inducing IL-6 production (Tosato and Jones, 1990; Cahill and Rogers, 2008; Tisoncik et al., 2012). Mice deficient in IL-1β displayed no levels of circulating IL-6 in response to turpentine (Zheng et al., 1995). Interleukin-1β can also modulate the production of IL-6 through STAT3 and NF-κB-dependent signaling pathways and involves acute phase proteins produced by the liver (Bode et al., 2012). This demonstrates that the NF-κB pathway is quite involved in the production of inflammatory cytokines and that targeting this pathway could be of therapeutic benefit at multiple levels: IL-1β production, IL-6 production, and IL-1β-induced IL-6 production.

Moreover, mice infected with IC-deficient rSARS-CoV E exhibited reduced levels of inflammasome-activated IL-1β in their lungs (Nieto-Torres et al., 2014). This reduction in IL-1β was accompanied by reduced levels of TNF and IL-6, demonstrating the importance of the E protein in the induction of an aberrant inflammatory response in SARS-CoV mice that contributes to the development of a cytokine storm, and ultimately culminates in ARDS. A recent preprint, published by Xia et al. (2020), reported that SARS-CoV-2 E alone was sufficient to induce a cytokine storm that caused damage similar to that observed in ARDS, both *in vitro* and *in vivo*. Notably, SARS-CoV-2 E induced IL-1β, IL-6, and TNF-α production resulting in histopathological features consistent with ARDS as observed in the spleen and lungs of mice. Inhibition of the E protein IC activity significantly reduced IL-1β, IL-6, and TNF-α production, corroborating its importance in COVID-19 immunopathology.

## Discussion and Conclusion

Despite the importance of the hCoV E protein, it is still poorly characterized and quite understudied. And although much progress has been made in hCoV research, the novelty of SARS-CoV-2 clearly leaves much still to be answered. The sequence similarity between the E proteins of SARS-CoV and SARS-CoV-2 strongly suggests the conservation of its functional characteristics (IC activity and PBM), thereby serving nearly identical purposes in the pathogenesis of COVID-19. Admittedly, a great divergence exists in the amino acid sequences of the E protein between the different CoV groups and, to an extent, within some of the groups. But the overall features and functions of the CoV E still remain largely intact (Masters, 2006). The importance of the E protein is evident by its involvement in the pathogenesis of SARS-CoV, and possibly SARS-CoV-2, making it an ideal therapeutic candidate. Already, a p38 MAPK inhibitor has shown promise in mice by alleviating the inflammation-induced symptoms brought on by the SARS-CoV E protein. Given the involvement of hCoV E in various aspects of the coronaviral life cycle, targeting E could hold the potential to stopping the spread of infection while simultaneously alleviating the symptoms and managing complications such as ARDS in severe SARS-CoV infections. Coronaviral research would certainly benefit from investigating the therapeutic potential of a p38 MAPK inhibitor in a SARS-CoV-2 infection of mice. The gravity of the COVID-19 pandemic warrants more research into hCoVs and how such outbreaks can be addressed, now more than ever.

Currently, vaccine and antiviral research are being done at a near-unprecedented rate, but while an effective countermeasure might only be available in as soon as 12 months, the hCoV pandemic continues to have a significant impact on people all over the world. The SARS-CoV E protein is paramount to the pathogenesis of the SARS disease as rSARS-CoV-ΔE viruses show no excessive inflammatory response and spare mice from immune-mediated lung damage. Our paper proposes the use of immunomodulatory or anti-inflammatory drugs that specifically target the already well-characterized inflammatory pathways activated by SARS-CoV E. Given the importance of IL-1β and IL-6 in the development of ARDS, drugs that expressly target IL-1β and IL-6 could lead to more favorable patient outcomes and reduce the rising mortality rate of COVID-19 while vaccine and antiviral research continue.

Amid the global rise in the mortality rate of COVID-19, effective management of inflammation and the cytokine storm, the crucial features of ARDS, should be of considerable priority. The use of the IL-6 receptor blocker, tocilizumab effectively reversed the cytokine storm in acute lymphocytic anemia (Grupp et al., 2013; Barrett et al., 2014). Tocilizumab has, accordingly, been suggested for use in the treatment of severe COVID-19, where Xu et al. (2020) has reported some promise in severe COVID-19 patients (Giamarellos-Bourboulis et al., 2020; Zhang C. et al., 2020). Already, blocking IL-1β activity in a broad array of inflammatory diseases has shown reduced disease severity and a reduction in the burden of disease (Dinarello, 2011). Inhibitors of IL-1 typically include the IL-1 receptor antagonist (Anakinra), the soluble decoy receptor (Rilonacept), and the anti-IL-1β monoclonal antibody (Canakinumab) (Schlesinger, 2014). The efficacy of rilonacept and canakinumab has even garnered approval by pharmaceutical companies, making such IL-1-directed therapies deserving of study as potential treatments to manage severe cases of COVID-19 (Dinarello et al., 2012).

The cellular pathways that lead to IL-1β and IL-6 production are well-characterized and could also serve as valuable therapeutic targets. A p38 MAPK pathway inhibitor led to an 80% survival rate of rSARS-CoV-infected mice, showing both the relevance of this pathway in SARS infections and the potential of this inhibitor in successfully managing severe cases of COVID-19 (Jimenez-Guardeño et al., 2014). Furthermore, inhibition of the Janus kinase/signal transducer and activator of transcription (JAK-STAT) pathway by ruxolitinib is effective in the treatment of hemophagocytic lymphohistiocytosis, a hyperinflammatory condition also characterized by a cytokine storm (Maschalidi et al., 2016). The JAK-STAT pathway is a common signal transduction pathway involved in the expression of many other cytokines also responsible for the immune-mediated damage of ARDS typical of severe SARS cases. Accordingly, this pathway can also be a target for blocking multiple cytokines simultaneously.

The importance of the hCoV E protein and its associated pathways is also demonstrated in the potential of a SARS-CoV-2 vaccine that lacks an E protein. Without the E protein to induce a cytokine storm, and subsequent complications like ARDS, undesired side-effects will be limited, while the vaccine still confers the necessary protection. Some studies have already demonstrated the potential of developing rSARS-CoV-ΔE vaccines, or ones with a mutated E protein to limit pathogenesis while still conferring the needed protection against a viral challenge after vaccination (DeDiego et al., 2007; Lamirande et al., 2008; Regla-Nava et al., 2015). Vaccines based on rSARS-CoV-ΔE retain their immunogenicity and efficacy, developing robust cellular and humoral immune responses and are effective despite an impaired ability to replicate in the host. One study even showed that a rSARS-CoV-ΔE-based vaccine can protect both young and aged mice, with no clinical disease observed in mice of any ages (Fett et al., 2013). The authors, however, cautioned prudence in the design of such vaccines, highlighting the need to possibly introduce additional mutations to enhance safety due to the recombinatory nature of CoVs (Masters, 2006; Perlman and Netland, 2009).

Admittedly, data on CoV E is sparse, but it should not reflect negatively on the importance of the protein in hCoV infections, especially not in the case of serious ones such as SARS-CoV-2. On the contrary, the importance of the E protein should, instead, underpin the need for more research in an effort to limit any likelihood of a future outbreak, possibly a more severe one. If there is anything to learn from the SARS, MERS, and COVID-19 outbreaks, it is that we do not know when they will happen nor what the nature of the outbreak will be.

## Author Contributions

BF conceptualized the manuscript. DS prepared the original draft. Both authors drafted, read, edited, and approved the final manuscript.

## Conflict of Interest

The authors declare that the research was conducted in the absence of any commercial or financial relationships that could be construed as a potential conflict of interest.

## Supplementary Material

FIGURE S1 Multiple sequence alignment (MSA) of the envelope (E) protein amino acid sequences from the seven human coronaviruses (hCoVs). The alignment was done using the Clustal OMEGA algorithm set at default parameters and performed and visualized with Jalview software (v 2.11.0). Topological domains (blue), the transmembrane domain (brown), and the PDZ-binding motif (PBM) (orange) are indicated. The PBM of SARS-CoV is confirmed, whereas the PBM for other hCoVs are putatively indicated. Accession IDs for hCoV-229E (P19741), hCoV-NL63 (Q6Q1S0), SARS-CoV (P59637), SARS-CoV-2 (QHD43418.1), MERS-CoV (K9N5R3), hCoV-OC43 (Q04854), and hCoV-HKU1 (Q5MQC8).

TABLE S1 Pairwise alignment indicating the percentage (%) similarity of the envelope (E) protein between the seven existing human coronaviruses (hCoVs) after multiple sequence alignment (MSA). The alignment was done using the Clustal OMEGA algorithm set at default parameters and performed and visualized with Jalview software (v 2.11.0). Accession IDs for hCoV-229E (P19741), hCoV-NL63 (Q6Q1S0), SARS-CoV (P59637), SARS-CoV-2 (QHD43418.1), MERS-CoV (K9N5R3), hCoV-OC43 (Q04854), and hCoV-HKU1 (Q5MQC8). hCoVs are grouped according to genera. a-CoVs: hCoV-229E and hCoV-NL63; b-CoVs: SARS-CoV, SARS-CoV-2, MERS-CoV, hCoV-OC43, hCoV-HKU1.
