# Supplementary figures and images for: Is There a Link Between the Pathogenic Human Coronavirus Envelope Protein and Immunopathology? A Review of the Literature
Source: Front Microbiol. 2020 Sep 3;11:2086. doi: 10.3389/fmicb.2020.02086 (PMC7496634; doi:10.3389/fmicb.2020.02086)

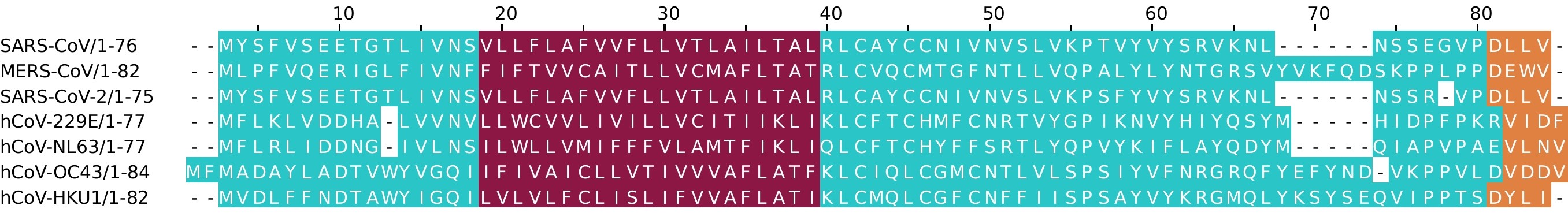

Supplement: FIGURE S1 — Multiple sequence alignment (MSA) of the envelope (E) protein amino acid sequences from the seven human coronaviruses (hCoVs). The alignment was done using the Clustal OMEGA algorithm set at default parameters and performed and visualized with Jalview software (v 2.11.0). Topological domains (blue), the transmembrane domain (brown), and the PDZ-binding motif (PBM) (orange) are indicated. The PBM of SARS-CoV is confirmed, whereas the PBM for other hCoVs are putatively indicated. Accession IDs for hCoV-229E (P19741), hCoV-NL63 (Q6Q1S0), SARS-CoV (P59637), SARS-CoV-2 (QHD43418.1), MERS-CoV (K9N5R3), hCoV-OC43 (Q04854), and hCoV-HKU1 (Q5MQC8). [file Image_1.JPEG]
