# Supplementary material for: Is There a Link Between the Pathogenic Human Coronavirus Envelope Protein and Immunopathology? A Review of the Literature
Source: Front Microbiol. 2020 Sep 3;11:2086. doi: 10.3389/fmicb.2020.02086 (PMC7496634; doi:10.3389/fmicb.2020.02086)
Supplement: TABLE S1 — Pairwise alignment indicating the percentage (%) similarity of the envelope (E) protein between the seven existing human coronaviruses (hCoVs) after multiple sequence alignment (MSA). The alignment was done using the Clustal OMEGA algorithm set at default parameters and performed and visualized with Jalview software (v 2.11.0). Accession IDs for hCoV-229E (P19741), hCoV-NL63 (Q6Q1S0), SARS-CoV (P59637), SARS-CoV-2 (QHD43418.1), MERS-CoV (K9N5R3), hCoV-OC43 (Q04854), and hCoV-HKU1 (Q5MQC8). hCoVs are grouped according to genera. a-CoVs: hCoV-229E and hCoV-NL63; b-CoVs: SARS-CoV, SARS-CoV-2, MERS-CoV, hCoV-OC43, hCoV-HKU1. [file Table_1.docx]

Supplementary Material

| **hCoVs** | *229E* | *NL63* | *SARS-CoV* | *SARS-CoV-2* | *MERS* | *OC43* | *HKU1* |
| --- | --- | --- | --- | --- | --- | --- | --- |
| *229E* |  | 46.75 | 23.68 | 25.64 | 26.25 | 22.62 | 28.05 |
| *NL63* | 46.75 |  | 18.42 | 18.42 | 21.95 | 25.97 | 26.25 |
| *SARS-CoV* | 23.68 | 18.42 |  | 94.74 | 35.90 | 19.23 | 25.00 |
| *SARS-CoV-2* | 25.64 | 18.42 | 94.74 |  | 34.67 | 22.78 | 26.67 |
| *MERS* | 26.25 | 21.95 | 35.90 | 34.67 |  | 28.05 | 23.17 |
| *OC43* | 22.62 | 25.97 | 19.23 | 22.78 | 28.05 |  | 52.44 |
| *HKU1* | 28.05 | 26.25 | 25.00 | 26.67 | 23.17 | 52.44 |  |
